# Suspected Type III Hypersensitivity Post-Inactivated Vaccination Leukocytoclastic Vasculitis in Two Sows

**DOI:** 10.3390/vetsci13080824

**Published:** 2026-08-18

**Authors:** Jean-François Da-Costa, Arnaud Lebret, Gwenaël Boulbria, Valérie Normand, Justine Favrel, Mathieu Brissonnier, Nadia Amenna-Bernard, Sophie Labrut, Marion Mosca, Didier Pin

**Affiliations:** 1 Porc.Spective Swine Vet Practice, 56920 Noyal-Pontivy, France; a.lebret@porc.spective.fr (A.L.); g.boulbria@porc.spective.fr (G.B.); v.normand@porc.spective.fr (V.N.); j.favrel@porc.spective.fr (J.F.); m.brissonnier@porc.spective.fr (M.B.); 2 Labocea, 22440 Ploufragan, France; nadia.amenna-bernard@labocea.fr (N.A.-B.); sophie.labrut@labocea.fr (S.L.); 3 Interactions Cells Environment, VetAgro Sup, Université de Lyon, 69280 Marcy l’Etoile, France; marion.mosca@vetagro-sup.fr (M.M.); didier.pin@vetagro-sup.fr (D.P.)

**Keywords:** type III hypersensitivity, vasculitis, vaccine adverse effect

## Abstract

This case report describes the investigation of two cases of cutaneous vasculitis in sows following vaccination. The herd was porcine reproductive and respiratory syndrome virus (PRRSV)-stable, with routine PRRSV-1 and porcine circovirus type 2 (PCV2) sows’ vaccination. Two sows developed red-to-purple macules on the udder, flanks, and hind limbs, occurring two and three days after vaccination, respectively. Diagnostic investigations included biochemistry, complete blood count, PCR, immunohistochemistry (IHC), serology, histopathology, Gram staining, and immunofluorescence (IF) to assess the involvement of type III hypersensitivity. Clinical examination and herd evaluation revealed no significant abnormalities. Both sows showed biochemistry results within the reference intervals. One sow had lymphocytosis. All PCR PCV2, PCV3 and PRRSV-1/2 on serum and IHC PCV2 conducted on skin biopsies were negative, although both animals were seropositive for PCV2. Histopathology revealed necrotizing leukocytoclastic vasculitis, and Gram staining detected no bacteria. IF failed to identify immune complex or complement deposition. However, type III hypersensitivity could not be excluded, as immune complexes are transient and biopsies were collected about 48 h after lesion onset. Overall, the findings support a vaccine-related adverse reaction. Vaccine adverse effects should therefore be considered in the differential diagnosis of cutaneous vasculitis in swine.

## Simple Summary

In a farrow-to-finish farm, two sows developed red-to-purple cutaneous lesions localised on the udder, flanks, and hind limbs. The aim of this study was to investigate the origin of these lesions. The lesions appeared a few days after the sows were vaccinated. Clinical examination and laboratory findings were not consistent with bacterial or viral infectious etiology. Immunofluorescence was performed to detect antibodies and complement deposits in order to investigate a hypersensitivity reaction. No antibody or complement deposits were detected. Nevertheless, a vaccine-induced adverse reaction associated with hypersensitivity remains the most plausible explanation, as immune complexes are transient and may no longer be detectable at the time of sampling.

## 1. Introduction

Hypersensitivity reactions are defined by exacerbated immune responses classified into four main types. Type III hypersensitivity involves immune complex formation and complement activation, leading to vascular injuries, especially vasculitis. Lesions can appear immediately or several days after the antigen exposure. In human medicine the antigen triggering the type III hypersensitivity may be of infectious origin, or non-infectious origin like drugs or vaccines [1]. Several vaccines has been suspected of causing vasculitis (Varicella-Zoster virus, influenza and COVID-19 vaccines) [2,3]. In pig production, vasculitis lesions are regularly triggered by infectious causes: porcine circovirus type 2 (PCV2) and 3 (PCV3), *Betaarterivirus suid* 1 and 2, also known as porcine reproductive and respiratory syndrome virus type 1 (PRRSV-1) and type 2 (PRRSV-2), *Actinobacillus* sp., *Erysipelothrix rhusiopathiae*, *Escherichia coli*, *Glaesserella parasuis*, *Leptospira interrogans*, *Salmonella* sp., *Streptococcus suis* and *Treponema pedis* [4,5,6,7]. Vasculitides of non-infectious origin are less documented and no vaccine used in pig production mentions any cases of vasculitis in their summary of product characteristics.

This clinical case aims to investigate two cases of leukocytoclastic vasculitis following vaccination of sows occurring in the absence of detectable infectious triggers. The vaccines suspected of causing vasculitis in this case were Rhiniseng^®^ (Laboratorios Hipra S.A., Spain), Suigen Rota Coli^®^ (Virbac, France) and Respiporc Flu 3^®^ (CEVA santé Animale, France). All three vaccines were inactivated vaccines. However, adjuvants and excipients differ from one another (except sodium chloride).

## 2. Case Presentation

### 2.1. Anamnesis and Clinical Examination

The case report concerns two sows of a 600-sow farrow-to-finish farm located in Brittany, France. The herd follows batch-production practices with farrowings scheduled every 2 weeks with a weaning age of approximately 21 days. Biosecurity measures are very well implemented according to French regulation. Regarding PRRSV-1, the herd is supposed to be positive stable with vaccination without evidence of sow-to-piglet transmission and no viral circulation on the finishing stage. The vaccination protocol is presented in Table 1.

**Figure 1:**
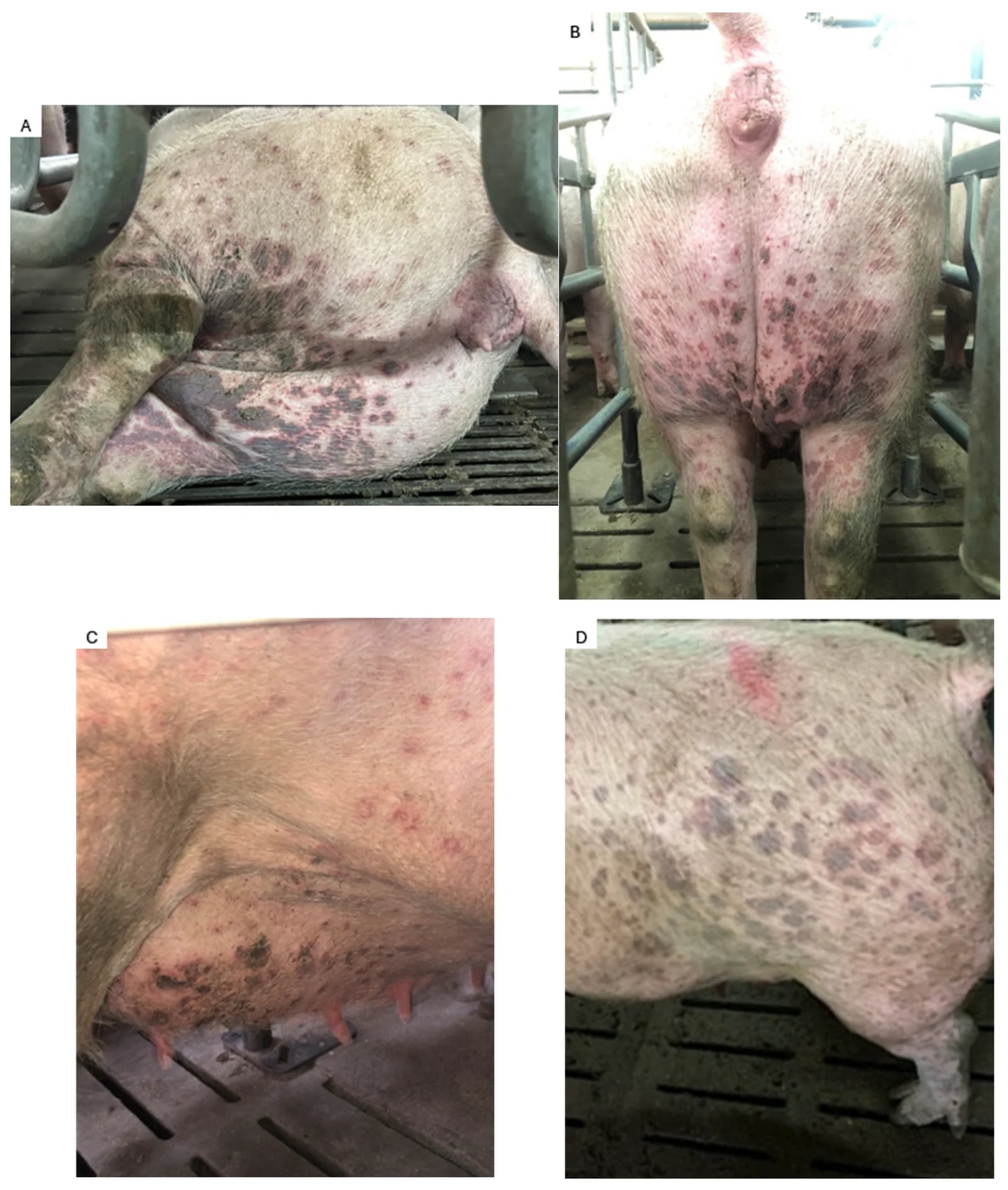
Red-to-purple macules localised on the hind limbs, udder and flanks of sow 1 (photos (**A**,**B**)) and sow 2 (photos (**C**,**D**)).

Seven weeks before farrowing, the first sow (Sow 1) received the rhinitis vaccine. Six hours later, she developed fever (40 °C) and anorexia for 2 days. Two days after the vaccination, multifocal, well-circumscribed, slightly raised and non-suppurative, red-to-purple macules occurred. They were not pruritic and ranged in diameter from 1 to 2 cm. They were localised to the udder, flanks and hind limbs, where they tended to coalesce (Figure 1). A clinical examination was carried out two days after macules appeared. The multiparous sow (parity 5) was in good body condition with a normal breathing pattern and normothermia. The sow demonstrated no reluctance to move and a good appetite.

Five months later, the second sow (Sow 2) received swine influenza vaccine, colibacillosis and rotavirus vaccines on the same week. Three days after swine influenza vaccination and five days after colibacillosis and rotavirus vaccination, sow 2 developed similar skin lesions (Figure 1). No hyperthermia was noticed by the farmer. The clinical examination was carried out two days after macules appeared. Sow 2 was a parity 8 and the clinical examination’s result was similar to sow 1.

At the herd level, the fertility rate was normal (92%) as was prolificacy (16.8 piglets born alive per litter). Stillbirth and mummification rates were stable (respectively 6.1% and 2.4%). No abortion was noticed.

### 2.2. Samples Collection

For both sows, all samples were taken two days after the skin lesions occurred, after the clinical examination.

Blood samples were taken from the external jugular vein with an 18G x 1 1/2” needle in dry, heparin and EDTA tubes.

Biochemistry assessments were conducted on the heparin tubes. Urea (UR3825, Randox^®^, Ireland), creatinine (CR3814, Randox^®^, Ireland), total protein (TP3869, Randox^®^, Ireland), albumin (AB3800, Randox^®^, Ireland), calcium (CA3871, Randox^®^, Ireland) and phosphorus (PH3872, Randox^®^, Ireland) were assessed, using colorimetric tests with a RX IMOLA device (Randox^®^, Ireland). Serum amyloid A was measured using ELISA assay (Serum Amyloid A TP802, Tridelta^®^, Ireland) and read with a SUNRISE device (Tecan^®^, Switzerland).

The complete blood count was performed on EDTA blood samples using Vet abc Plus+ system (Scil^®^, Germany), based on impedance and spectrophotometric measurements. Blood smears were stained using the RAL 555 kit.

Extraction methods, automated extraction systems, PCR reagents and PCR instruments used for qPCR PCV2, PCV3 and RT-PCR PRRSV-1/PRRSV-2 are presented in Table 2.

PCV2 serologies were performed with the ELISA Ingezim Circo IgG (11.PCV.K.1/2, Ingenasa^®^, Spain) test.

Urine samples were collected from sow 1 during a spontaneous voiding in a sterile sample container with boric acid. No urine was collected from sow 2 due to the practical impossibility to do it. A urinary analysis was performed for sow 1 by macroscopic and microscopic examination using a Kova^®^ slide (Glasstic slide 10, Kova^®^, USA) and a urinary stick (Multistix 8 SG, Siemens^®^, USA).

Punch biopsies were done on the edge of 2 macules for both sows. Biopsies were kept in a 4% formaldehyde solution respecting a 1 to 10 volume ratio at room temperature.

For histopathological examination, formalin-fixed samples were embedded in paraffin wax, cut into 3 µm sections and stained with haematoxylin, eosin and saffron. Two to three cut levels per sample were evaluated and examined using a Leica DM 2500 microscope (Leica Microsystems^®^, Germany). Pictures were taken using an XCD U100CR digital camera (Sony^®^, Japan) and registered using the Archimed imaging software (version 12.3.11).

For immunohistochemistry (IHC), 4 μm thick sections of all tissues were cut and placed on silane-coated slides (SuperFrost Plus, Thermofisher^®^, USA). IHC was performed with a monoclonal rabbit anti-PCV2 antibody (in-house antibody produced by Anses Ploufragan, France) at 1/200 concentration.

Immunofluorescences (IF) were conducted to detect IgG, IgM, IgA, C3. Antibody references used are presented in Table 3. Antibodies were used at different concentrations (1/100, 1/50 and 1/20). Slides were read with a fluorescent microscope using a filter adapted to fluorochrome (Zeiss^®^, France). Analysis was performed with ZEN software (version ZEN 2). Slides incubated with PBS solution instead of antibodies served as negative controls.

All samples were directly sent to an external laboratory (LABOCEA, Ploufragan, France) and kept in positive cold (2° to 8 °C) during transportation. Biochemistry was assessed in LABEO (Normandy, France), urinary analysis, qPCR PCV2, RT-PCR PRRSV1/PRRSV-2, histolopathological examination, IHC and PCV2 serology were assessed by LABOCEA (Ploufragan, France). qPCR PCV3 was conducted by Anses Ploufragan-Plouzané-Niort laboratory (Ploufragan, France). Direct immunofluorescence (DIF) and Gram staining were conducted by VetAgro Sup laboratory (Marcy l’Etoile, France).

**Table 3:** Antibody references used for IF.

Antibody	Reference
IgG	Goat anti-Pig IgG (H + L), FITC (PA1-84850, Invitrogen^®^, USA)
IgM	Goat anti-Pig IgM, FITC (NBP2-36762, Novus^®^, USA)
IgA	Goat anti-Pig IgA, FITC (NB726, Novus^®^, USA)
C3	Rabbit polyclonal anti-C3 Pig (BS-6416R, Bioss^®^, USA) and Goat Anti-rabbit IgG, FITC (65-6111, Invitrogen^®^, USA)

## 3. Results

### 3.1. Biochemistry and Haematological Findings

For both sows, biochemistry values were within the reference ranges, particularly the blood urea and creatinine concentrations (Table 4).

Haematological findings are presented in Table 5. A slight increase in MCHC was observed for both sows but was considered of doubtful clinical significance, as it was isolated and not associated with other haematological abnormalities. In the absence of leukocytosis, the slight variations in the proportions of lymphocytes, monocytes, neutrophils and basophils in sow 1 and of monocytes and basophils in sow 2, compared with the reference intervals, are unlikely to be clinically significant.

### 3.2. Urinary Analysis

The urinary examination for sow 1 is presented in Table 6. The urine specific gravity (1.010) was within the reference interval (1.010–1.050) [12]. Crystals were observed but not red blood cells or leukocytes. The urinary pH was within the reference interval (5.5–7.5) [4].

### 3.3. PCV2-, PCV3- and PRRS-Related Investigations

All serum samples tested negative for PCV2 and PCV3 DNA and PRRSV-1/PRRSV-2 RNA. PCV2 was not detected by IHC (Table 7).

### 3.4. Histological, Gram Staining and Immunofluorescence Examination

**Figure 2:**
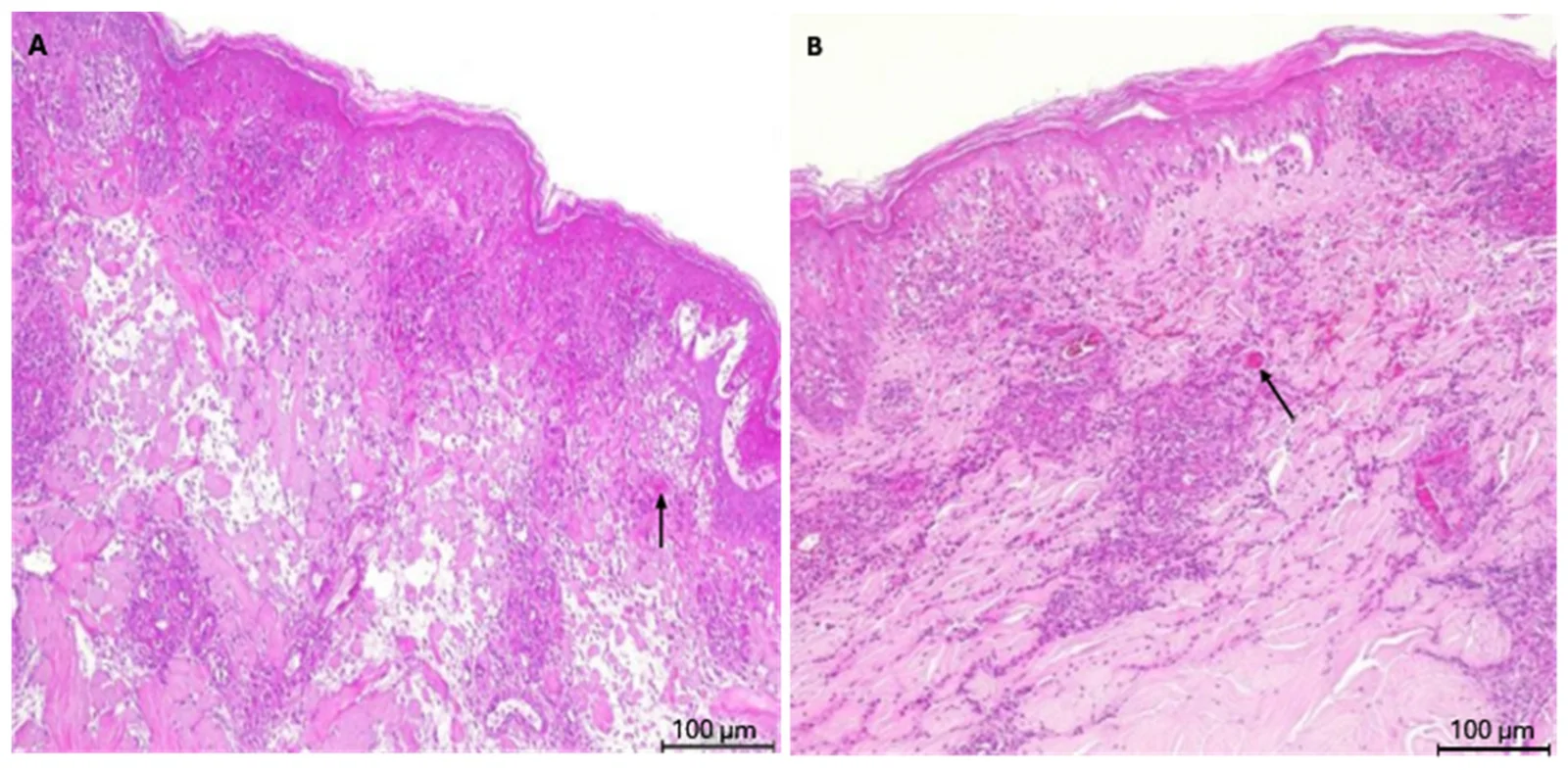
Histopathological skin lesion HE staining showing necrotizing vasculitis (arrows) for sow 1 (**A**) and sow 2 (**B**). Scale bar: 100 µm.

Histological examination of the punch biopsies revealed mild epithelial hyperplasia and mild to moderate infiltration of the perivascular and vascular walls by hypersegmented neutrophils in both sows. Some eosinophils were also present in the perivascular and vascular walls of sow 2. Mild necrotising vasculitis and fibrinoid degeneration, characterised by perivascular fibrinous exudates and cell debris, were also observed (Figure 2). The upper dermis and mid-dermal blood vessels were affected.

**Figure 3:**
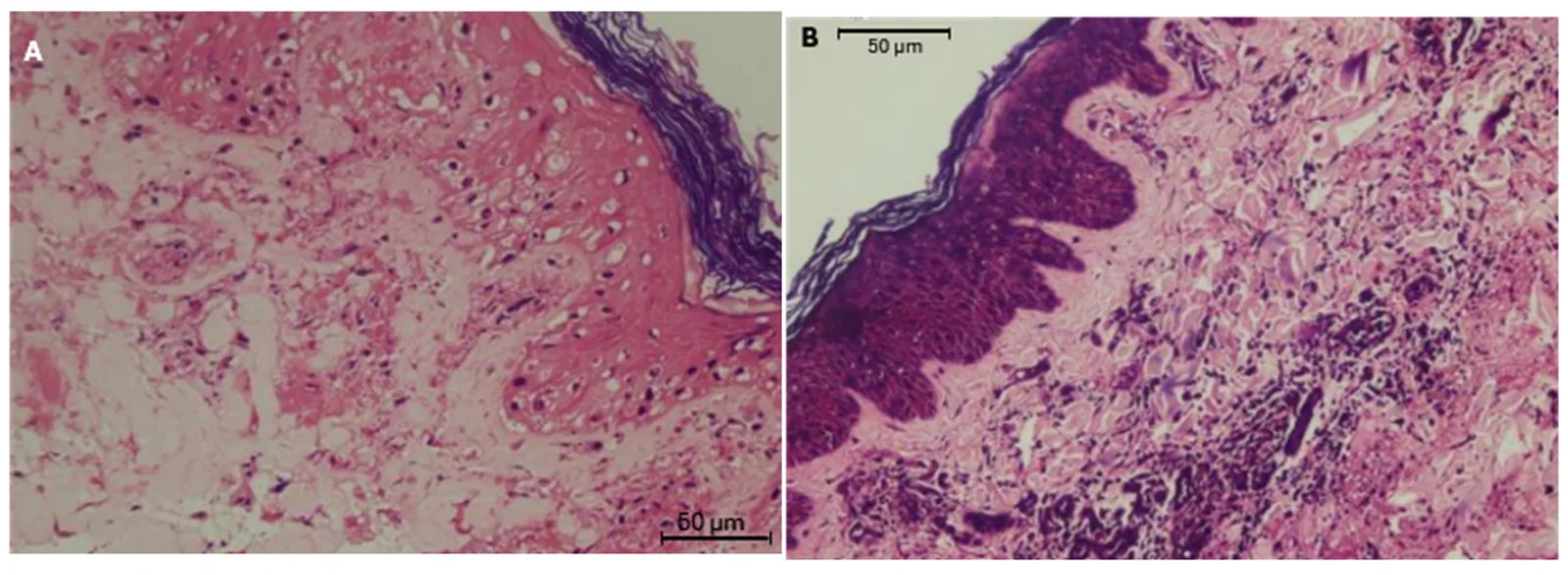
Gram staining on skin biopsies for sow 1 (**A**) and sow 2 (**B**). Magnification of the image: (**A**) ×400 and (**B**) ×200. No bacteria observed. Scale bar: 50 µm.

Skin biopsy Gram staining was negative for both sows (Figure 3). The observation of several slides of each biopsy at a magnification of ×1000 did not reveal the presence of bacteria.

**Figure 4:**
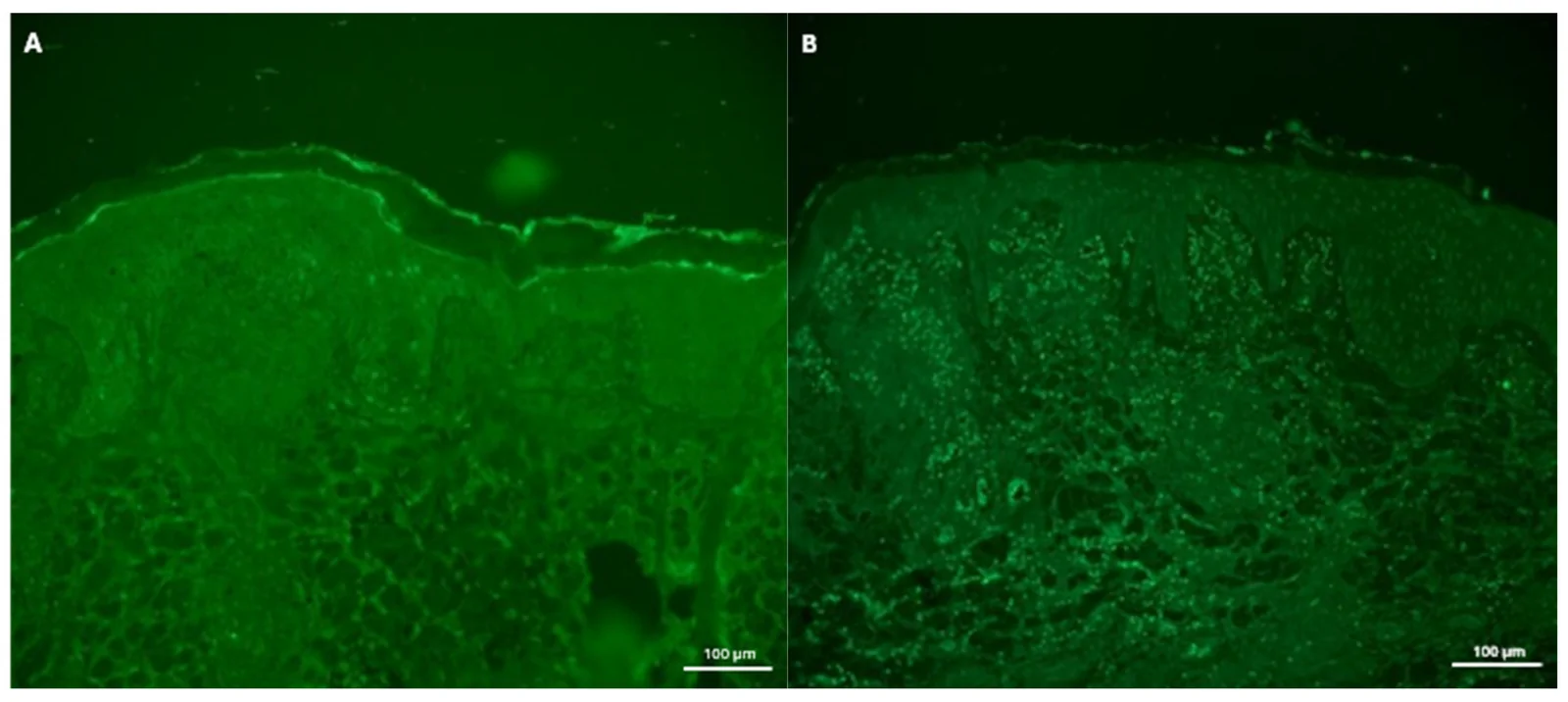
Skin biopsies direct immunofluorescence using rabbit polyclonal anti-C3 pig and Goat Anti-rabbit IgG, FITC for sow 1 (**A**) and sow 2 (**B**). No immunofluorescence signal detected. Scale bar: 100 µm.

Direct immunofluorescence did not detect IgG, IgM, IgA or C3 deposition (Figure 4).

Both sows received amoxicillin (Amoxipro injectable, Ceva Santé Animale, Libourne, France) 15 mg/kg intramusculary and ketoprofen (Rifen 100 mg/mL, Vetviva Richter GMBH, Wels, Austria) the day skin lesions appeared and 48 h after. Papules and macules regressed within one week after their appearance for sow 1. Two days after treatment, scabs replaced some lesions for sow 2.

The sows farrowed 6 weeks after vaccination. Parturition and prolificacy were normal for both.

## 4. Discussion

Post-vaccination reaction is commonly described in the vaccine’s summary of product characteristics (section adverse events) used in pig production. In our case report, a few days after vaccination, two sows developed a purpuric macular eruption. The clinicopathological findings were consistent with dermal leukocytoclastic vasculitis. Infectious causes such as PCV2, PCV3, PRRSV-1/PRRSV-2 and bacteria were considered unlikely based on laboratory results, absence of systemic signs and normal renal function.

Similar cases have been reported in humans. Cutaneous reactions were delayed and occurred several days after vaccination [3,13]. Even though cutaneous reactions occurring at a distance from the injection are rare, vasculitis have been described after Varicella-Zoster virus, influenza and COVID-19 vaccination [2,13]. In human medicine, no risk factors have been identified in patients who developed post-vaccination vasculitis [14]. Missoum et al. [3] described one case with leukocytoclasic vasculitis lesions developing after SARS-CoV-2 inactivated vaccination. The vaccine used in this human case was an aluminum hydroxide-adjuvanted vaccine. Leukocytoclastic vasculitis was observed on histopathological examination of skin lesions and plasma protein C level increased 9 days after vaccination. The patient suffered from a renal failure characterised by hyperuremia and hypercreatininaemia. In our case, the two sows presented leukocytoclastic vasculitis too and a normal SAA level without renal failure at sampling time (four days and five days after the last vaccination for sow 1 and sow 2, respectively). Similarities between humans’ lesions after SARS-CoV-2 vaccination and sows’ lesions after vaccination may suggest a similar cause. The three vaccines suspected to be involved in the sows’ vasculitis were all inactivated vaccines. However, their compositions differ. Among them, only Rhiniseng^®^ is adjuvanted with aluminum hydroxide as with the human case mentioned above. Differences in vaccine composition did not allow the identification of any specific component that could have contributed to the development of vasculitis in sows.

Infection causes were thoroughly investigated. A bacteria-induced vasculitis was considered less likely as sow 2 exhibited no additional clinical signs suggestive of systemic infection (no fever, no anorexia, clinically healthy). Several bacterial pathogens have been associated with vasculitis in pigs, including *Actinobacillus* sp., *Erysipelothrix rhusiopathiae*, *Escherichia coli*, *Glaesserella parasuis*, *Leptospira interrogans*, *Salmonella* sp., *Streptococcus suis* and *Treponema pedis* [5,6,7]. In our case, bacterial culture on skin biopsies could not be carried out, but Gram staining showed no bacteria on the slides although this staining might not highlight all the aforementioned bacteria. Main viral pathogens causing vasculitis in pigs are circoviruses and PRRSV-1/PRRSV-2 [4]. Vasculitis associated with PCV2 or PCV3 has been well described in porcine dermatitis and nephropathy syndrome (PDNS) [15,16,17]. Affected pigs are usually depressed, prostrate and anorexic. Mortality approaches 100% in animals over three months of age due to renal failure caused by glomerulonephritis [4,18]. Sipos et al. [19] also reported elevated urea and creatinine values in PDNS-affected pigs compared to controls. In our cases, biochemistry and urine analysis did not reveal kidney lesions. Although isolated skin lesions may occur in rare instances, PDNS-affected herds generally display additional circovirus-associated diseases [16,20]. No such conditions were observed in the herd before or after the present cases. In addition, qPCR for PCV2, PCV3 and RT-PCR PRRSV-1/PRRSV-2 as well as PCV2 IHC were all negative, which did not support a viral etiology. While PCV2 DNA may be inconsistently detected in skin lesions, it is typically present in serum from PDNS-affected pigs [21] which was not the case here.

DIF was performed to investigate a potential hypersensitivity reaction following vaccination. No IgG, IgM, IgA or C3 deposition was detected in the skin lesions. In the SARS-CoV-2 post-vaccination vasculitis case described by Missoum et al. [3], skin immunofluorescence was negative too.

This case has potential limitations. Bacteriology on skin biopsies could have been conducted to explore bacterial origin. During our initial investigations, we found this cause unlikely, which is why all skin biopsies sampled were fixed in formaldehyde solution. Subsequently, when investigating potential bacterial causes, we decided to carry out Gram staining. However, in the absence of systemic reactions such as hyperthermia, an infectious origin is unlikely.

Concerning direct immunofluorescence, no signal was detected. The delay between the lesion onset and sample collection could have influenced the results. Indeed, Thibault et al. [22] conducted DIF in suspected PDNS cases. C3 and IgM signals were the two most detected signals. However, these signals were no longer detected a few days after the onset of clinical lesions, supporting that antigen–antibody complex could be transient. In human medicine, skin biopsy had to be sampled rapidly (within 48 h of lesion onset) because leukocytoclastic vasculitis tends to evolute in non-specific lymphocytic perivascular lesions after 48 h [23,24]. In the present cases, biopsies were sampled approximately 48 h after skin lesions appeared, which may explain the absence of detectable immune deposits. Moreover, little is known about the sensitivity and specificity of IF in pigs. A lack of sensitivity could also explain the absence of detectable signals.

## 5. Conclusions

Vasculitis has not been previously described as an adverse reaction to any vaccines used in this herd. Nevertheless, in the absence of an identifiable infectious pathogen after differential diagnosis was done, a vaccine-associated hypersensitivity reaction remains the most plausible explanation for the vasculitis observed in these sows. Therefore, vaccine-induced adverse effects should be included in the differential diagnosis of porcine dermal vasculitis.

## Figures and Tables

**Table 1:** Gilts and sows vaccination protocol.

Trade Name	Manufacturer	Target Pathogen(s)	Gilts	Sows
Circoflex^®^	Boehringer Ingelheim Vetmedica GmbH, Ingelheim am Rhein, Germany	Porcine circovirus type 2	1 and 4 weeks after arrival (AA) 4 weeks before farrowing (BF)	Mass vaccination every 16 weeks
**Respiporc Flu 3^®^**	**CEVA santé Animale, Libourne, France**	**Influenza A virus (H3N2, H1N1, H1N2)**	**2 and 5 weeks AA**	**Mass vaccination every 16 weeks**
Porcilis Ery Parvo^®^	Intervet, Beaucouze, France	Porcine parvovirus + *Erysipelothrix rhusiopathiae*	1 and 4 weeks AA	2 weeks after farrowing (AF)
**Suigen Rota Coli^®^**	**Virbac, Carros, France**	**Neonatal diarrhoea (rotavirus type A, ** ***Escherichia coli *** **F4/F5/F6/F41 and ** ***Clostridium. Perfringens*** ** type A/C)**	**7 and 3 weeks BF**	**3 weeks BF**
Enteroporc Coli AC^®^	CEVA santé Animale, Libourne, France	Neonatal diarrhoea (*Escherichia coli* F4/F5/F6 and *Clostridium Perfringens* type A/C)	6 and 2 weeks BF	2 weeks BF
**Rhiniseng^®^**	**Laboratorios Hipra S.A., Amer, Spain**	**Atrophic rhinitis (** ***Bordetella bronchiseptica*** ** and ** ***Pasteurella multocida*** **)**	**3 and 6 weeks AA**	**7 weeks BF**
Porcilis PRRS^®^	Intervet, Beaucouze, France	PRRSV-1	1 and 4 weeks AA	6 weeks BF and 1 week AF
Autogenous vaccine	Labocea, Ploufragan, France	*Streptococcus suis* and *Salmonella typhimurium* and *Glaesserella parasuis*	2 and 5 weeks AA	5 weeks BF

Vaccines in bold correspond to vaccines suspected of causing vasculitis in our case. The names in italics correspond to the bacterial species names.

**Table 2:** Extraction methods, automated extraction system, PCR reagents and PCR instruments used for qPCR PCV2, PCV3 and RT-PCR PRRSV-1/PRRSV-2.

	qPCR PCV2	qPCR PCV3	RT-PCR PRRSV-1/PRRSV-2
Extraction methods	Indispin QIAcube Pathogen Kit, Indical Biosciences^®^, Germany	QIAamp DNA Mini Kit, Qiagen^®^, Netherlands	MagMax CORE 2.0, Thermofisher^®^, USA
Automated extraction systems	QIAcube Qiagen^®^, Netherlands	Manual extraction	FLEX96, Thermofisher^®^, USA
PCR reagents	Anses in-house reactive, France	TaqMan Master Mix, Thermofisher^®^, USA Anses in-house primer, France (modified according to Franzo et al. [8])	VetMax PRRSV EU/NA 2.0, Thermofisher^®^, USA
Real-time PCR systems (PCV2, PCV3) or Thermal cycler (PRRS)	CFX96, Biorad^®^, USA	QuantStudio 5 Real-Time PCR, Thermofisher^®^, USA	CFX96, Biorad^®^, USA

**Table 4:** Biochemistry findings.

	Sow 1	Sow 2	Reference Interval ^1^
Urea (mmol/L)	2.8	2.3	2.3; 5.8 [9]
Creatinine (µmol/L)	181	198	160; 325 [9]
Total protein (g/L)	82.9	89.5	65.3; 89.7 [9]
Albumin (g/L)	36.2	41.1	33.4; 47.9 [9]
Serum amyloid A (SAA) (µg/mL)	32.72	57.25	31.25; 500 ^2^
Calcium (mmol/L)	2.52	2.58	1.4; 3.3 [5]
Phosphore (mmol/L)	2.82	2.52	1.4; 3.3 [9]

^1^ Reference interval based on 2.5th to 97.5th interpercentile range for gestating sows [9] and growing pigs [5]; ^2^ Tridelta SAA multispecies kit range.

**Table 5:** Haematological findings.

	Sow 1	Sow 2	Reference Interval ^1^
Red blood cells (10^12^/L)	4.8	5.62	4.3; 7.2 [10]
White blood cells (10^9^/L)	14.8	15.2	7.6; 18.8 [10]
Thrombocytes (10^9^/L)	207	203	90.7; 400.7 [10]
Hematocrit (%)	0.317	0.365	0.31; 0.49 [10]
Hemoglobin (10^9^/L)	104	125	94; 145 [10]
MCV (fL)	66	65	61; 75 [10]
MCHC (g/L)	329	341	291; 318 [10]
Lymphocytes (%)	56	40	17.3; 40.5 [10]
Monocytes (%)	2	8	3.3; 6.8 [10]
Neutrophils (%)	30	49	47.7; 72.0 [10]
Eosinophils (%)	12	8	2.9; 15.7 [10]
Basophils (%)	0	0	0.5; 1.4 [11]

^1^ Reference interval with 90% confidence limits for gestating sows.

**Table 6:** Sow 1 urinary examination by macroscopic and microscopic examination and urinary stick.

	Results
Appearance ^1^	Clear
Leukocytes ^2^	<10,000/mL
Red blood cells ^2^	None
Crystals ^2^	Some calcium oxalate and multiple urates
Leukocytes ^3^	None
Nitrite ^3^	None
Protein ^3^	None
pH ^3^	6
Blood ^3^	None
Density ^3^	1.01
Ketones ^3^	None
*Glucose* ^3^	None

^1^ Evaluated by macroscopic examination. ^2^ Evaluated by microscopic examination. ^3^ Evaluated by urinary stick (SIEMENS^®^ Multistix 8 SG).

**Table 7:** IHC PCV2 and qPCR PCV2, PCV3 and RT-PCR PRRSV results for both sows.

	IHC PCV2	qPCR PCV2	qPCR PCV3	RT-PCR PRRSV
Sow 1	Negative	Negative	Negative	Negative
Sow 2	Negative	Negative	Negative	Negative

## Data Availability

The original contributions presented in this study are included in the article. Further inquiries can be directed to the corresponding author.
